# Supplementary material for: Important Lessons on Long-Term Stability of Amino Acids in Stored Dried Blood Spots
Source: Int J Neonatal Screen. 2023 Jun 21;9(3):34. doi: 10.3390/ijns9030034 (PMC10366855; doi:10.3390/ijns9030034)
Supplement: Supplementary file 1 [file IJNS-09-00034-s001.zip › IJNS-2429660-supplementary.pdf]

## Supplementary materials

**Table S1.** Retention times, m/z transition and MS settings of the (stable isotope labeled) amino acids.

| Amino acid | Internal standard | Ret.time (min) | MRM transition (m/z) | DP/CE[1] |
|---|---|---|---|---|
| glycine / $^{13}C_2{}^{15}N$-glycine | | 5.7 | 76->76 / 79->79 | 25/5 |
| alanine / $^{13}C_3{}^{15}N$-alanine | | 5.2 | 90->44 / 94->47 | 25/18 |
| serine / $^{13}C_3{}^{15}N_3$-serine | | 6.2 | 106->60 / 110->63 | 40/15 |
| proline / $^{13}C_5{}^{15}N$-proline | | 4.0 | 116->70 / 122->75 | 30/20 |
| threonine / $^{13}C_4{}^{15}N$-threonine | | 5.5 | 120->74 / 125->78 | 30/20 |
| valine / $^{13}C_5{}^{15}N$-valine | | 4.0 | 118->72 / 124->77 | 30/13 |
| leucine / $^{13}C_6{}^{15}N$-leucine | | 2.9 | 132->86 / 139->92 | 30/13 |
| isoleucine / $^{13}C_6{}^{15}N$-isoleucine | | 3.1 | 132->86 / 139->92 | 30/13 |
| aspartate/ $^{13}C_4{}^{15}N$-aspartate | | 7.2 | 134->74 / 139->77 | 30/20 |
| glutamate / $^{13}C_5{}^{15}N$-glutamate | | 6.4 | 148->84 / 154->89 | 30/16 |
| histidine / $^{13}C_6{}^{15}N_3$-histidine | | 7.4 | 156->110 / 165/118 | 30/20 |
| lysine / $^{13}C_6{}^{15}N_2$-lysine | | 7.9 | 147->84 / 155->90 | 25/18 |
| phenylalanine / $^{13}C_9{}^{15}N$-phenylalanine | | 2.8 | 166->120 / 176->129 | 30/18 |
| tyrosine / $^{13}C_5{}^{15}N$-tyrosine | | 4.2 | 182->136 / 192->145 | 30/20 |
| arginine/$^{13}C_6{}^{15}N_4$-arginine | | 7.6 | 175->116 / 185->122 | 30/20 |
| citrulline / D4-citrulline | | 6.5 | 176->159 / 180->163 | 30/18 |
| glutamine/ $^{13}C_3{}^{15}N$-alanine | | 6.1 | 147->84 / 94->47 | 30/17 |
| ornithine / $^{13}C_6{}^{15}N_2$-lysine | | 8.0 | 133->70 / 155->90 | 35/14 |
| taurine / $^{13}C_3{}^{15}N$-alanine | | 4.3 | 126->108 / 94->47 | 58/20 |
| hydroxyproline / $^{13}C_5{}^{15}N$-proline | | 4.2 | 132->86 / 122->75 | 20/21 |
| tryptophan / $^{13}C_9{}^{15}N$-phenylalanine | | 2.9 | 205->188 / 176->129 | 30/17 |
| asparagine / $^{13}C_3{}^{15}N$-alanine | | 11.5 | 133->74 / 94->47 | 25/18 |
| cystine / $^{13}C_6{}^{15}N_2$-cystine | | 13.8 | 241->152 / 249->156 | 30/18 |
| methionine / $^{13}C_5{}^{15}N$-methionine | | 9.8 | 150->104 / 156 -> 109 | 30/13 |

retention times are represented in minutes, MRM transitions (multiple reaction monitoring) indicate first transition/second transition and quantifying/qualifying used. DP= declustering potential; CE= collision energy.

**Table S2.** Limits of detection and quantification, intra-and inter-assay precision of 23 amino acids in the control samples.

| | | | DBS | | | | | | Low commercial QC | | | | | | Vendor Range | high commercial QC | | | | | | Vendor range |
|---|---|---|---|---|---|---|---|---|---|---|---|---|---|---|---|---|---|---|---|---|---|---|
| **Amino acid** | **LOD** | **LOQ** | **intra-assay CV (%)** | | | **inter-assay CV (%)** | | | **intra-assay CV (%)** | | | **inter-assay CV (%)** | | | | **intra-assay CV (%)** | | | **inter-assay CV (%)** | | | |
| | **µM** | **µM** | **Mean (µM)** | **SD** | **CV (%)** | **Mean (µM)** | **SD** | **CV (%)** | **Mean (µM)** | **SD** | **CV (%)** | **Mean (µM)** | **SD** | **CV (%)** | **µmol/L** | **Mean (µM)** | **SD** | **CV (%)** | **Mean (µM)** | **SD** | **CV (%)** | **µmol/L** |
| **alanine** | 2.4 | 8.1 | 405.2 | 26.9 | 6.6 | 402.2 | 4.7 | 1.2 | 779.0 | 43.3 | 5.6 | 822.9 | 65.6 | 8.0 | 460-955 | 1638.0 | 101.9 | 6.2 | 1711.4 | 122.0 | 7.1 | 1007-1869 |
| ***arginine*** | 3.7 | 12.3 | 27.1 | 2.2 | 8.3 | 17.9 | 3.0 | 17.0 | 13.6 | 1.9 | 13.8 | 9.3 | 4.1 | **43.7** | 4.90-23.1 | **206.7** | 15.6 | 7.5 | **37.9** | 12.1 | **32.0** | 44.5-178 |
| **asparagine** | 1.6 | 5.4 | 72.7 | 2.5 | 3.5 | 82.4 | 2.8 | 3.4 | 63.9 | 4.6 | 7.1 | 51.7 | 9.4 | 18.1 | * | 62.0 | 3.5 | 5.6 | 49.2 | 10.5 | 21.4 | * |
| ***aspartate*** | 4.3 | 14.2 | 31.6 | 9.3 | **29.5** | 30.6 | 3.5 | 11.3 | 32.2 | 6.7 | **20.9** | **20.2** | 6.3 | **31.0** | 21.4-64.1 | 211.0 | 32.0 | 15.2 | 184.0 | 41.3 | 22.5 | 128-299 |
| **citrulline** | 1.6 | 5.3 | 31.0 | 2.5 | 8.2 | 29.4 | 4.1 | 13.9 | 28.1 | 1.4 | 4.9 | 22.7 | 4.9 | 21.5 | 13.8-36.3 | 305.9 | 14.5 | 4.7 | 286.4 | 45.0 | 15.7 | 108-370 |
| ***glutamine*** | 3.3 | 11.0 | 566.4 | 62.4 | 11.0 | 448.9 | 5.3 | 1.1 | 69.7 | 6.0 | 8.6 | 5.7 | 2.9 | **50.0** | * | 68.4 | 5.4 | 7.9 | 17.7 | 6.7 | **38.2** | * |
| **glutamate** | 0.4 | 1.2 | 103.7 | 7.0 | 6.7 | 80.7 | 4.8 | 6.0 | 732.7 | 21.8 | 3.0 | 713.1 | 82.4 | 11.6 | 416-772 | **1196.0** | 70.5 | 5.9 | 1096.4 | 153.8 | 14.0 | 638-1186 |
| **glycine** | 3.6 | 12.0 | 259.2 | 24.9 | 9.6 | 227.5 | 13.1 | 4.7 | 595.2 | 45.8 | 7.7 | 607.4 | 61.9 | 10.2 | 380-705 | 1577.1 | 405.2 | 9.3 | 1699.7 | 142.5 | 8.4 | 967-1796 |
| **histidine** | 2.8 | 9.3 | 88.9 | 12.6 | 14.2 | 75.3 | 3.0 | 3.9 | 117.3 | 11.3 | 9.6 | 83.1 | 13.3 | 16.0 | * | 105.0 | 5.5 | 5.2 | 82.9 | 14.0 | 16.9 | * |
| ***hydroxyproline*** | 0.5 | 1.6 | 11.4 | 7.0 | **61.2** | 11.3 | 4.1 | **36.3** | 22.7 | 8.8 | **38.6** | 21.8 | 7.9 | **36.2** | * | 19.5 | 7.3 | **37.7** | 23.9 | 7.9 | **33.2** | * |
| **isoleucine** | 0.9 | 2.8 | 52.6 | 3.6 | 6.9 | 53.5 | 2.5 | 4.8 | 107.4 | 5.5 | 5.1 | 106.7 | 7.1 | 6.7 | * | 113.8 | 10.4 | 9.2 | 119.6 | 10.0 | 8.3 | * |
| **leucine** | 2.8 | 9.3 | 120.0 | 7.4 | 6.2 | 119.4 | 10.8 | 9.1 | 225.5 | 8.3 | 3.7 | 234.2 | 22.2 | 9.5 | 162-301 | 728.1 | 61.7 | 8.5 | 763.4 | 43.3 | 5.7 | 462-859 |
| ***lysine*** | 0.9 | 2.9 | 162.6 | 8.6 | 5.3 | 90.4 | 5.3 | 5.9 | 232.9 | 12.5 | 5.4 | 93.9 | 32.5 | **34.6** | * | 229.2 | 11.0 | 4.8 | 95.6 | 33.4 | **34.9** | * |
| **methionine** | 2.5 | 8.5 | 22.9 | 2.1 | 9.4 | 24.3 | 0.9 | 3.8 | 38.1 | 2.4 | 6.3 | 36.0 | 3.3 | 9.2 | 27.6-57.2 | 399.5 | 24.9 | 6.2 | 405.5 | 20.6 | 5.1 | 256-531 |
| ***ornithine*** | 3.5 | 11.5 | 72.7 | 2.5 | 3.5 | 84.2 | 25.2 | **30.0** | **63.9** | 4.6 | 7.1 | **109.3** | 42.1 | **38.5** | 125-292 | **62.0** | 3.5 | 5.6 | 321.7 | 112.8 | **35.1** | 267-623 |
| **phenylalanine** | 2.1 | 7.1 | 60.6 | 5.6 | 9.2 | 59.1 | 2.0 | 3.4 | 132.2 | 9.9 | 7.5 | 132.4 | 14.7 | 11.1 | 70.3-164 | 849.4 | 56.6 | 6.7 | 837.2 | 59.6 | 7.1 | 447-1043 |
| **proline** | 1 | 3.2 | 208.1 | 9.9 | 4.8 | 227.4 | 14.1 | 6.2 | 279.6 | 12.2 | 4.4 | 298.7 | 24.7 | 8.3 | 184-342 | 595.2 | 31.3 | 5.3 | 620.6 | 31.5 | 5.1 | 376-698 |

| | | | | | | | | | | | | | | | | | | | | | | |
|---|---|---|---|---|---|---|---|---|---|---|---|---|---|---|---|---|---|---|---|---|---|---|
| **serine** | 4.4 | 14.7 | 103.8 | 8.5 | 8.2 | 110.5 | 21.4 | 19.3 | 267.9 | 19.5 | 7.0 | 262.6 | 25.0 | 9.5 | * | 248.2 | 19.3 | 7.8 | 247.6 | 34.0 | 13.7 | * |
| **taurine** | 7.2 | 24.0 | 215.2 | 16.4 | 7.6 | 170.9 | 6.7 | 3.9 | 163.6 | 17.1 | 10.5 | 151.3 | 22.6 | 14.9 | * | 155.1 | 14.6 | 9.4 | 134.5 | 18.9 | 14.0 | * |
| **threonine** | 1.8 | 6.0 | 130.1 | 14.1 | 10.9 | 126.9 | 0.8 | 0.7 | 193.9 | 11.4 | 5.9 | 188.6 | 16.1 | 8.5 | * | 183.0 | 18.3 | 10.0 | 179.1 | 11.1 | 6.2 | * |
| **tryptophan** | 2.9 | 9.7 | 47.4 | 4.3 | 9.0 | 41.1 | 5.9 | 14.5 | 63.1 | 3.7 | 5.9 | 52.8 | 8.9 | 16.9 | * | 59.5 | 4.9 | 8.2 | 49.0 | 8.8 | 18.0 | * |
| **tyrosine** | 3.4 | 11.4 | 116.6 | 9.0 | 3.8 | 72.7 | 2.1 | 2.8 | 116.6 | 9.0 | 7.7 | 108.7 | 6.4 | 5.9 | 50.7-134 | 715.7 | 40.3 | 5.6 | 698.6 | 59.4 | 8.5 | 335-781 |
| **valine** | 1.8 | 6.0 | 207.8 | 17.7 | 8.5 | 240.9 | 10.4 | 4.3 | 272.7 | 15.3 | 5.6 | 293.8 | 22.5 | 7.7 | 122-323 | 682.8 | 53.8 | 7.9 | **<u>774.7</u>** | 55.2 | 7.1 | 330-770 |

LOD= limit of detection. calculated as 3.3*standard deviation (SD)/ slope of the calibration curve; LOQ= limit of quantification. calculated as 10*SD/slope of calibration curve; CV= coefficient of variation. calculated as the ratio of the SD to the mean (sd/mean*100%.). The inter-assay precision per amino acid. calculated from quality control samples (n = 30 in ten experiments). Mean concentrations for Arginine and Glutamine in the low QC samples were below the limit of quantification (in. bold. italic. underlined). Underlined and bold AA in the table have intra- and/or interassay CV >25% in at least one of the QC samples. Bias compared to the established concentrations by the vendor is presented underlined and bold.

*No established range was supplied for this amino acid by the vendor

**Table S3.** Mann-Whitney U for the annual percentile changes in controls compared to deceased children

| Amino acid | P-value | Amino acid | P-value |
|---|---|---|---|
| Alanine | 0.834 | Lysine | 0.548 |
| Arginine | <0.01 | Methionine | 0.640 |
| Aspargine | 0.690 | Ornithine | 0.841 |
| Aspartate | <0.05 | Phenylalanine | 0.095 |
| Citrulline | <0.05 | Proline | 0.295 |
| Glutamine | 0.908 | Serine | 0.402 |
| Glutamate | <0.05 | Taurine | 0.295 |
| Glycine | 0.310 | Threonine | 0.642 |
| Histidine | <0.05 | Tryptophan | 0.920 |
| Hydroxyproline | 0.841 | Tyrosine | 0.660 |
| Isoleucine | <0.05 | Valine | 0.295 |
| Leucine | 0.421 | | |

*Mann-Whitney U test were done to compare annual percentile changes in DBS AA concentrations of controls and deceased children. Most annual percentile changes in AA between groups were comparable. Some AA showed different annual percentile change rates. Upon plotting (see Figure S1). these deviations were seen to be attributed to the control group. probably due to the loss of power from the smaller sample size.*

**Figures: Plotting of annual percentile changes in AA concentrations between controls and deceased children**

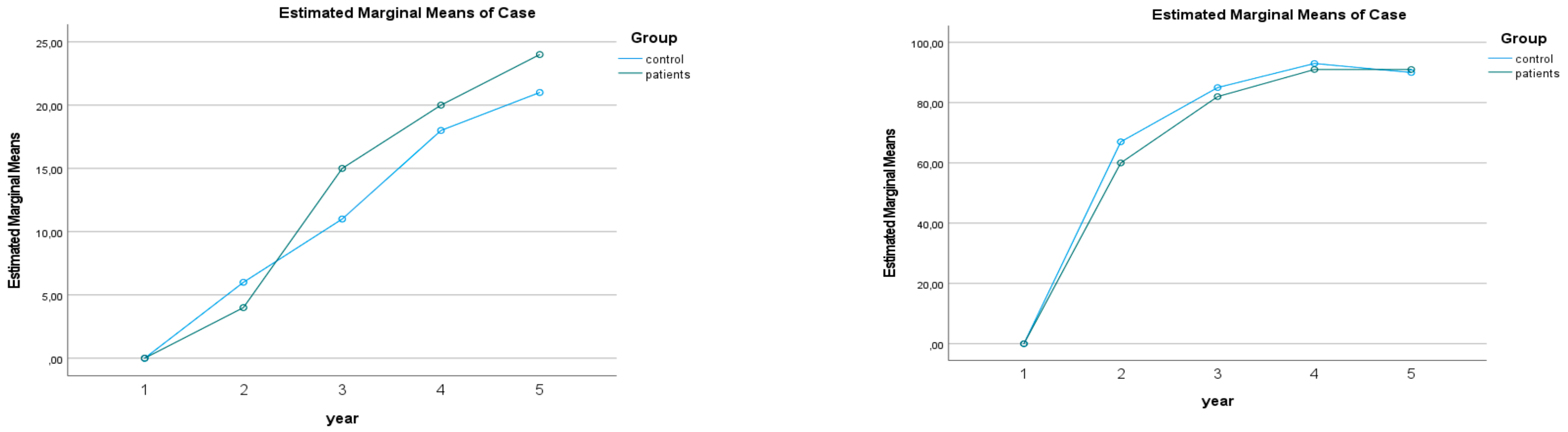


**Figure S1. Examples of good agreement. Leucine and Glutamine**

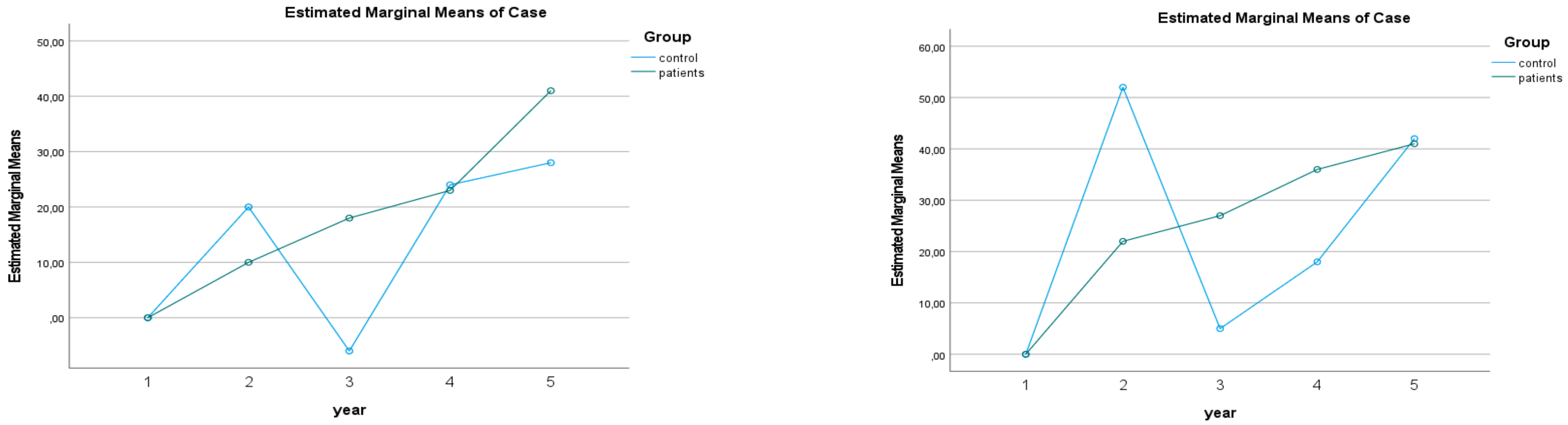


**Figure S2. Examples of poor agreement. Citrulline and Arginine**
